# Influenza A(H7N7) Virus among Poultry Workers, Italy, 2013

**DOI:** 10.3201/eid2208.160246

**Published:** 2016-08

**Authors:** Simona Puzelli, Caterina Rizzo, Concetta Fabiani, Marzia Facchini, Paolo Gaibani, Maria P. Landini, Carlo Gagliotti, Maria L. Moro, Roberto Rangoni, Luisa Loli Piccolomini, Alba C. Finarelli, Marco Tamba, Giovanni Rezza, Silvia Declich, Isabella Donatelli, Maria R. Castrucci

**Affiliations:** Istituto Superiore Sanità, Rome, Italy (S. Puzelli, C. Rizzo, C. Fabiani, M. Facchini, G. Rezza, S. Declich, I. Donatelli, M.R. Castrucci);; St. Orsola University Hospital, Bologna, Italy (P. Gaibani, M.P. Landini);; Agenzia Sanitaria Regionale Emilia-Romagna, Bologna Region (C. Gagliotti, M.L. Moro, R. Rangoni, L.L. Piccolomini, A.C. Finarelli);; Istituto Zooprofilattico Sperimentale della Lombardia e dell’Emilia Romagna, Brescia, Italy (M. Tamba)

**Keywords:** influenza A(H7N7) virus, influenza virus, avian influenza virus, viruses, influenza, respiratory infections, poultry, poultry workers, outbreak, serologic investigation, Italy

**To the Editor:** In August 2013, an outbreak of infection with highly pathogenic influenza A(H7N7) virus occurred in Emilia-Romagna, Italy, and >1 million birds were culled (*1*). Prevention measures were immediately applied, and all workers involved in culling activities wore personal protective equipment (PPE), including face masks with eye protection. These workers were monitored for clinical symptoms, and 3 workers with laboratory-confirmed cases of conjunctivitis caused by infection with influenza A(H7N7) virus were reported during the 3-week outbreak (*2*). Workers did not receive chemoprophylaxis.

A serologic study was conducted in December 2013 to identify potential asymptomatic infections following exposure to influenza A(H7N7) virus. This study was approved by the ethics committee of the Istituto Superiore di Sanità (protocol no. PRE787/13CE13/401).

A total of 93 of 140 workers directly involved in culling activities, including the 3 confirmed case-patients with conjunctivitis, participated in the study. All participants completed a questionnaire that obtained information for demographics, poultry exposure, and use of PPE.

Paired acute-phase and convalescent-phase serum samples were available only for the 3 H7 subtype–positive persons with conjunctivitis. We tested these paired serum samples and single serum samples obtained from virus-exposed workers for antibodies against influenza A(H7N7) virus strain A/Italy/3/2013 (*2*) by using hemagglutination inhibition (HI) and microneutralization (MN) assays (*3*,*4*). Other H7 subtype viruses previously circulating in Italy were included in the analysis to rule out potential cross-reactivity with influenza A(H7N7) virus (*5*). HI titers >10 and MN titers >20 were considered positive; only HI-positive serum samples confirmed 3 times by MN assay were considered positive results for influenza A(H7N7) virus.

We detected antibodies against influenza A(H7N7) virus in convalescent-phase serum samples from the 3 H7 subtype-positive patients and 2 asymptomatic persons but found no seropositivity against other H7 subtype viruses (Table). Because of lack of acute-phase serum samples, we could not assess whether seropositivity for the 2 asymptomatic persons, 1 (RA32) of whom worked with poultry before the outbreak, was caused by infection acquired during the outbreak. All workers were trained and most participants, including the 2 asymptomatic influenza A(H7N7) virus–seropositive persons, reported that PPE was commonly used during culling on infected premises. Nevertheless, it is likely that worker compliance with PPE was not always 100% during the 3-week outbreak because of poor knowledge and real perception of biologic risks among workers.

**Table:** HI and MN antibody titers for influenza A(H7N7) virus and other H7 subtype viruses in serum samples of 5 men, Italy, 2013*

Person ID and phase type†	Age, y	Activity of person	Date of sample collection	Virus strain (subtype) and titer										
				A/It/3/2013 (H7N7)			A/Tk/It/3889/ 1999 (H7N1)			A/Tk/It/214845/ 2002 (H7N3)			A/Ck/It/2837-54/2007 (H7N3)	
				HI	MN		HI	MN		HI	MN		HI	MN
1														
Acute	51	PW, culling	Sep 6	10	<10		<10	<10		<10	<10		<10	<10
Convalescent			Dec 6	**20**	**35**		<10	<10		<10	<10		<10	<10
2														
Acute	46	Culling	Sep 6	10	<10		<10	<10		<10	<10		<10	<10
Convalescent			Dec 11	**20**	**62**		<10	<10		<10	<10		<10	<10
3														
Acute	49	Culling	Sep 7	<10	<10		<10	NT		<10	NT		<10	NT
Convalescent			Dec 23	**10**	**87**		<10	<10		<10	<10		<10	<10
FO10‡	34	Culling	Dec 23	**20**	**72**		<10	<10		<10	<10		<10	<10
RA32‡	55	PW, culling	Dec 11	**20**	**33**		<10	<10		<10	<10		<10	<10

*Bold indicates titers of seropositive persons (HI positive results confirmed 3 times by MN). Values for 1 of 3 MN assays that showed similar results are reported. Seropositive persons were selected from 93 persons who participated in the study among 140 persons involved in culling activities. HI, hemagglutination inhibition; ID, identification; MN, microneutralization; NT, not tested; PW, poultry worker. †Persons 1, 2, and 3 had laboratory-confirmed cases of conjunctivitis caused by infection with influenza A(H7N7) virus. ‡Asymptomatic person.

Future efforts should ensure timely collection of paired serum samples from all workers involved in avian influenza outbreaks, especially when infections occur in humans. Strict compliance with recommended preventive control measures and serologic surveillance programs are crucial to avoid and eventually assess risk for infections with avian influenza viruses in persons exposed to infected poultry.
